# Tuberculosis

**Published:** 1905-09

**Authors:** J. W. Hurt

**Affiliations:** Atlanta, Ga.


### TUBERCULOSIS.

BY J. W. HURT, M.D., ATLANTA, GA.

*Mr. President and Gentlemen:*

I have selected for my subject tonight Tuberculosis, not because it's new, but because it's old, and so old that not enough importance is given to it. When we consider that one-fourth of the deaths, barring accidents, are caused from tuberculosis, it is high time we were on the war-path fighting with every available man and means this white plague that is destroying so many human beings. It invades every portion of the human body, but has a selective affinity for the lungs, therefore pulmonary tuberculosis, or, in common parlance, consumption, is most common. It is the most prevalent disease in the world, and most terrible, since hope of recovery is so rarely confirmed that it is held to be incurable. It is infectious, and if it were as contagious as it is deadly, the world would soon be depopulated. Think for a moment, one in four sacrificed to this dread disease, and yet we believe it can be made to disappear from the world. Osler says few reach maturity and none old age without having had a focus of this disease. The germ is ubiquitous and none escape it. The resisting power of the human body is very great, hence all are not vanquished by this germ. But suppose, if you please, the resisting powers be below normal, the germs will then find a resting place in some weak spot, say in the apex of the right lung, the place most preferable and we begin our exit. It may be slow on account of our resisting powers, but sure and certain.

It is now the accepted theory that tuberculosis of the lungs is not hereditary, but acquired by inhalation of the tubercle bacillus, and that these bacilli are disseminated by the undestroyed sputum of those already infected. This sputum dried upon a cloth will retain its vitality for six months, and it is stated that the dust from the head-board of a consumptive bed will cause the disease when injected into a rabbit, and in a test case injection made from a decoction of the feathers of a consumptive's bed produced the disease in a guinea pig.

Professor Whittaker, one of the best authorities on tuberculosis, compares man to a test-tube inclosed in skin instead of glass and containing much more culture-soil. Like a test-tube he stands with open mouth and receives what falls into it from the air, hence the importance of destroying the sputum that dries and flying through the air looking for some fertile spot to lodge, take root, multiply and in a short while the grim reaper, death, calls to receive his own, and we are left to mourn a departed friend that we could not relieve. Consumptives are often found in dark, poorly ventilated rooms, where the tubercle bacillus loves to dwell unmolested by light and air, its common enemy. This is the reason it's most often found in the apex of the right lung, it being dark and less active.

The patient in the last stages, weak, emaciated, bloodless, not able to raise his head, expectorates upon the walls, bedding, old newspapers, or floor, and in a short time this sputum dries and flying through the air seeks other victims that chance to be in reach. The striking distance of the germs I do not know, but have seen blocks in the thickly tenement houses scourged by this plague.

This sputum contains colonies of the bacilli, and it is said that diluted four hundred thousand times and injected into a guinea pig will propagate the disease.

Flick says it depends almost entirely upon the house, it being the granary, and if it were not for the house it would soon have to disappear. Sunlight, air and water are its natural enemies. Then let's fight it with the weapons that nature has provided, assisted by some of our best germacides. The question is often asked to give some practical means of preventing inoculation of tuberculosis. This demand is upon us all. What a glorious thing it would be if we could give a prescription for so many pills to be taken after each meal and feel that they would relieve the patient; but not so, we hav'nt the remedy so simple. About all we can say is, don't, don't sleep with consumptives; don't kiss them; don't carry infection from hand to mouth; don't occupy a bed or room that has been used by a consumptive until it has been thoroughly disinfected; don't patronize a dairy having infected cattle. There are a few other don'ts that apply to the consumptive that are of vast importance to prevent them infecting others. Don't spit on the walls of your rooms; don't spit on the floor; don't spit on the sidewalks; don't drink from vessels that others drink from; don't empty your cuspidores on the streets without first disinfecting—above all, don't fail to burn the sputum.

The authorities place no restrictions upon consumptives. They are permitted to come and go at will, leaving a virulent trail of germs in their wake. If a case of smallpox was allowed the same freedom, people would rise up in arms; yet smallpox, even before the days of vaccination, killed only 20 to 30 per cent., while that of tuberculosis is practically complete. If the fight on tuberculosis was half so vigorous as the present fight on yellow fever we would soon conquer it and drive it from the face of the earth.

Yellow fever kills a very small proportion of the world's inhabitants, still people flee from it like grim death. The most rigid quarantines are enforced and the United States government takes charge, trying to stamp it out, and yet it is a curable disease and only a small per cent. of those attacked die. Then again there is only a small part of the world where yellow fever exists, whereas, the entire globe is subject to tuberculosis; and yet no municipality, county, state or government quarantines against tuberculosis. On the other hand, they establish hospitals in the cities, allowing the inmates to come and go at will. If these hospitals are to be established, as they should be, let them be located in the rural districts where the inmates can get sunshine, pnre air and water, and let them till the ground and live as much as possible out of doors and eat vegetables. It is an injustice to the patient and the outside world to locate these hospitals in a thickly populated district of a city or town where they are confined to the house or small lot. The inmates need sunshine, pure air and water and a plenty of latitude and longitude. Again, most consumptives are timid and do not care to expose themselves to the gaze of the public. They will therefore seclude themselves as much as possible in the hospital, taking very little exercise in the open sunshine, thereby hastening on to eternity.

Now, as to the people who live in the vicinity of this city hospital; they have rights to their homes for which they paid their money and they do not want such an institution for their neighbor. In the first place, it is anything but pleasant to be where you can see at all times incurable and suffering people. It mars the pleasure of home life, and homes should be cheerful and happy. Again, there is danger from infection. I do not know how far the germs can be carried through the air but do know they will live for six months or more under favorable circumstances. Be the hospital ever so careful some of these billions of germs will escape to the detriment of the people in the vicinity. These hospitals should be located in the rural districts in a hundred-acre field far removed from any farm-house or dwelling. I think every county or parish should have a medical society and a board of health, and in this country every state congressional district should have a consumptive hospital supported by the district and every case of tuberculosis should be reported by the physicians.

The poor who are not able to care for themselves should be sent to these hospitals, and in all cases the bedding and clothing burnt and the house thoroughly fumigated and disinfected. No one now holds to the old idea that consumption is hereditary, but infectious, and the reason so many die in the same family is because they are constantly exposed to the germs. We are the guardians of the people's health; it therefore becomes our duty to educate them as to hygiene, infection and contagion, gaining their confidence and getting their co-operation, and maintaining a vigorous fight on the lines suggested. I believe we can make a wonderful improvement in our mortuary report.

We are not living as long as we should. While the average is increasing, I think it should continue to increase until it will be fifty instead of thirty years. There is more in the prevention than the healing art, and if the sputum is the cause of consumption, then destroy the sputum, and we stop the disease. Our medical societies are getting better organized all over the country. In union there is strength, and under a thorough organization we can control legislation and have laws enacted that will be a blessing to the present and future generations. Let us stand together as one man, reorganizing our societies all over the State, and make a battle royal on this dreaded of all diseases and wipe it from our State and country. The object of my paper is not to give you something new on the disease, but to get you to thinking on the lines of hospitals, disinfecting and fumigating, and destroying the germs that take away so many of our loved ones. You may suggest that this idea of hospitals in every congressional district would be too expensive; that the Georgia Legislature would refuse to pass such a bill. What good is there in money if it is not for pleasure and comfort we derive from it, and what pleasure is there in life if we are not able to enjoy it? There are enough doctors in our State to educate the people on these lines, and we can easily bring them up by public speeches and newspaper articles to a point where we can demand of our representatives in the Senate and House some legislation that we think should be made for the general good, welfare and longevity of our people. I offer this hospital suggestion as the one that strikes me with most favor, and I wish a free discussion on the subject, and if any one can offer something better, I will be glad to hear from him.

Think on these lines, gentlemen, and let us do something for suffering humanity, and the future generations will rise up and call us blessed.

---

### CARROLL COUNTY, GA., MEDICAL ASSOCIATION.

The Carroll County Medical Association met in regular semi-monthly session in the headquarters of the organization in Carrollton, on August 15th, and in addition to other important business transacted, this association was made a unit in the Georgia Medical Association, and as such the officers are: President, Dr. J. C. Smith, of Sand Hill; Secretary-Treasurer, Dr. J. F. Cole, of Carrollton; Vice-President, Dr. J. D. Hamrick, of Carrollton. Dr. J. D. Hamrick, of Carrollton, was elected delegate from this organization to the State Association, which meets in Augusta next April.

